# Hepatitis B Virus Reactivation after Switch to Cabotegravir/Rilpivirine in Patient with Low Hepatitis B Surface Antibody 

**DOI:** 10.3201/eid3008.240019

**Published:** 2024-08

**Authors:** Eisuke Adachi, Ayako Sedohara, Kotaro Arizono, Kazuaki Takahashi, Amato Otani, Yoshiaki Kanno, Makoto Saito, Michiko Koga, Hiroshi Yotsuyanagi

**Affiliations:** The University of Tokyo, Tokyo, Japan

**Keywords:** hepatitis B, hepatitis B virus, HIV, HBV, antiretroviral therapy, cabotegravir, rilpivirine, HBV reactivation, vaccine-escape mutation, viruses, Japan

## Abstract

A patient in Japan with HIV began antiretroviral therapy because of acute hepatitis B virus (HBV) 15 years ago, with low hepatitis B surface antibody, and experienced breakthrough HBV reactivation 4 months after switching from bictegravir/emtricitabine/tenofovir alafenamide to cabotegravir/rilpivirine. An immune escape mutation, E164V, was identified in the isolated HBV DNA.

The 2013 guidelines outlined by the Centers for Disease Control and Prevention indicate in cases where hepatitis B surface antibody (anti-HBs) titers fall below the cut off value of 10.0 mIU/mL, administration of booster vaccinations is generally not required, barring certain exceptions, such as immunocompromised persons (*1*). This recommendation is based on the understanding that exposure to hepatitis B virus (HBV) typically triggers B lymphocytes, culminating in the production of adequate antibody levels within a short timeframe. However, the effectiveness of this antibody response following HBV exposure depends on the persons immune status. The primary goal in managing chronic hepatitis B is to achieve an undetectable hepatitis B surface antigen (HBsAg) level. Cases where seroconversion from HBsAg to anti-HBs occurs is considered a functional cure, although, the risk for de novo reactivation persists and is not eliminated (*2*).

Isolated hepatitis B core antibody (anti-HBc) positivity, defined as antigen negative, anti-HBs negative, and anti-HBc positive, is considered a risk factor for occult HBV infection (*3*) and de novo HBV reactivation (*4*), indicating 2 distinct pathogenic pathways. The first scenario involves HBsAg negativity resulting from treatment or spontaneous resolution, without subsequent seroconversion to anti-HBs. The second scenario occurs when anti-HBs levels decline below the threshold of detection after initially testing positive. When anti-HBs levels decline, exposure-induced boosting is expected to provide a preventive effect. 

We describe the case of a patient with HIV whose anti-HBs titers declined below the threshold of detection after an initial confirmation of anti-HBs positivity. The patient’s medications were changed from bictegravir/emtricitabine/tenofovir alafenamide (B/F/TAF) to cabotegravir/rilpivirine (CAB/RPV). His HBsAg positivity recurred, and he reverted to being an HBV carrier. 

Ethics approval was granted by the ethics board of the Institute of Medical Science, University of Tokyo (approval no. 2022-48-1128). The patient gave consent for publication in accordance with the policies of Emerging Infectious Diseases and the International Committee of Medical Journal Editors.

## The Case

A 52-year-old man with HIV had contracted an acute HBV infection 15 years earlier. Antiretroviral therapy (ART) consisting of tenofovir disoproxil fumarate/emtricitabine (TDF/FTC) and lopinavir/ritonavir (LPV/RTV) was initiated at the time, and the acute hepatitis rapidly improved. The patient’s liver enzymes normalized 2 months after beginning ART, and he tested negative for HBV DNA and HBsAg 4 months after beginning ART (Table 1). Anti-HBs titers were detected at 2.03 IU/mL 9 months after beginning ART, and it was believed that his acute hepatitis B did not transition into a chronic HBV infection. His anti-HBs titers were again detected at 18.6 IU/mL 6 years after the initial diagnosis of the acute HBV infection. There was no reactivation of HBV while on stable ART containing TDF/FTC or TAF/FTC. Two years after the confirmation of seroconversion, his anti-HBs levels decreased below the cutoff value but remained around 8.0 mIU/mL. He tested positive for anti-HBc. 

**Table 1:** Laboratory values over time of a person in Japan with HIV after diagnosis of an acute HBV infection, from diagnosis to 12 years*

Category	Time from HBV diagnosis							
	0 mo	1 mo	3 mo	9 mo	5 y	6 y	8 y	12 y
ART regimen	NA	TDF/FTC+LPV/RTV	TDF/FTC	TDF/FTC	TDF/FTC	TDF/FTC	TDF/FTC	B/F/TAF
Laboratory values								
Anti-HBs, IU/mL†	20.8	0.00	N/A	2.03	N/A	18.6	8.55	9.34
HBsAg, mIU/mL‡	119,533	68,065	552	0.00	0.00	0.00	0.00	0.00
Anti-HBe, inhibition %§	Negative	Negative	Negative	Negative	Negative	NA	NA	NA
HBeAg, S/CO¶	1,840	1,290	2.5	Negative	Negative	NA	NA	NA
HBV DNA, log copies/mL	>8.8	6.8	3.8	ND	ND	ND	NA	ND
ALT, U/L	418	937	8	17	23	17	57	66
Total bilirubin, mg/dL	0.3	0.5	0.5	0.5	0.7	0.3	0.4	0.4
HBcrAg, log U/mL#	NA	NA	NA	NA	NA	NA	NA	Negative

*ALT, alanine aminotransferase; anti-HBe, hepatitis B e-antibody; anti-HBs, hepatitis B surface antibody; ART, antiretroviral therapy; B/F/TAF, bictegravir/emtricitabine/tenofovir alafenamide; HBcrAg, hepatitis B core-related antigen; HBeAg, hepatitis B e-antigen; HBsAg, hepatitis B surface antigen; HBV, hepatitis B virus; LPV/RTV, lopinavir/ritonavir; mIU, milli-IU; NA, not applicable; ND, not detected; RPV, rilpivirine; S/CO, signal-to-cutoff ratio; TDF/FTC, tenofovir disoproxil fumarate/emtricitabine. †Cutoff value 10 IU/mL. ‡Cutoff value 10 mIU/mL. §Cutoff value 50% inhibition. ¶Cutoff value 1.0 S/CO. #Cutoff value 3.0 log U/mL.

After 14 years of ART, the patient expressed a preference to change to a long-acting injectable ART. We considered 3 additional factors before making this medication change: the patient did not become an HBV carrier after his acute hepatitis B diagnosis, his HBsAg and his HBV DNA had remained undetectable for >10 years, and his anti-HBs had declined only after initial positive confirmation. His hepatitis B core-related antigen, which is shown to remain positive longer than HBsAg and HBV DNA in acute hepatitis B infections (*5*,*6*), was negative. After a 1-month lead-in with oral CAB/RPV, his ART was changed to an injectable CAB/RPV. 

The patient’s liver enzyme levels increased to alanine aminotransferase (ALT) 103 U/L (reference range 4–44 U/L) 4 months after switching to CAB/RPV. Both his HBsAg and HBV DNA tested positive again, while the level of anti-HBs decreased to nearly zero (Table 2). The probability of reinfection with a novel HBV strain was considered low because his sexual partner was on ART containing tenofovir during this period and the patient denied any other potential exposures. The patient’s CD4 count was 512 cells/μL, indicating sufficient immune function. The patient’s liver enzymes returned to within reference ranges 4 months later, whereas the HBsAg and HBV DNA remained elevated. The patient’s ART regimen was switched to B/F/TAF 14 months after the change to CAB/RPV because of concerns of a concurrent chronic HBV infection. 

**Table 2:** Laboratory values over time of a person in Japan with HIV 2 years before changing ART regimen to CAB/RPV and the period while on CAB/RPV, showing HBV reactivation in month 4*

Category	Time from ART change								
	–2 y	0 mo	1 mo	2 mo	4 mo	6 mo	8 mo	10 mo	14 mo
ART regimen	B/F/TAF	CAB/ RPV oral	CAB/RPV injectable	CAB/RPV	CAB/RPV	CAB/RPV	CAB/RPV	CAB/RPV	B/F/TAF
Laboratory values									
Anti-HBs, IU/mL†	N/A	6.57	5.65	8.78	0.20	0.20	0.00	0.13	0.00
HBsAg, mIU/mL‡	0.00	0.00	0.00	0.00	816	90,332	124,890	143,785	131,765
Anti-HBe, inhibition %§	NA	NA	NA	Negative	6.16	31.1	54.4	56.0	55.3
HBeAg, S/CO¶	NA	NA	NA	Negative	60.5	404	680	835	915
HBV DNA, log copies/mL	ND	NA	NA	NA	3.9	NA	NA	NA	8.2
ALT, U/L	66	25	0	25	103	158	40	32	27
Total bilirubin, mg/dL	0.4	0.6	0.5	0.6	0.5	0.6	0.6	0.7	0.4
HBcrAg, Log U/mL#	NA	NA	Negative	NA	NA	NA	NA	NA	>7.1

*ALT, alanine aminotransferase; anti-HBe, hepatitis B e-antibody; anti-HBs, hepatitis B surface antibody; ART, antiretroviral therapy; B/F/TAF, bictegravir/emtricitabine/tenofovir alafenamide; CAB, cabotegravir; HBcrAg, hepatitis B core-related antigen; HBeAg, hepatitis B e-antigen; HBsAg, hepatitis B surface antigen; HBV, hepatitis B virus; NA, not applicable; ND, not detected; RPV, rilpivirine. †Cutoff value 10 IU/mL. ‡Cutoff value 10 mIU/mL. §Cutoff value 50% inhibition. ¶Cutoff value 1.0 S/CO. #Cutoff value 3.0 log U/mL.

We cloned 2 full-length HBV isolates and determined the genome sequences from serum samples obtained at 2 intervals: the first after 4 months of treatment with CAB/RPV, coinciding with the re-positivity of HBsAg, and the second 14 months after treatment with CAB/RPV (Appendix). Our genomic analysis revealed identical clones of genotype A2, and we observed no increases in mutations after reactivation (Table 3). Of note, E164V was identified within the S region and is recognized as a vaccine-escape mutation (Appendix Figure) (*7*). No drug resistance–associated mutations were observed in the polymerase region.

**Table 3:** Results of HBV DNA genome analysis on HBV strain isolated during hepatitis B reactivation in a person with HIV after changing antiretroviral therapy regimen to cabotegravir/rilpivirine*

Category	Region				
	S gene	P gene	Pre-S1/pre-S2/S	X gene	Precore/core
Position	155–835	2307–3221, 1–1623	2854–3221, 1–835	1374–1838	1814–2458/1901–2458
Size, bp	681	2538	1203	465	645
Mutations					
Vaccine-escape	E164V	NA	NA	NA	NA
Drug-resistant	NA	ND	NA	NA	NA
Promoter	NA	NA	NA	NA	ND
Other	NA	NA	ND	ND	T1858C,† T1674C†

*The full length of the HBV isolate was 3,221 bp. HBV, hepatitis B virus; NA, not applicable; ND, not detected. †These mutations are typically observed in genotype A2.

There are multiple explanations for the re-emergence of HBsAg in this case. The mostly likely explanation is our patient did not attain a functional cure of HBV. The reduction in HBsAg might have been because of ART containing TDF/FTC or TAF/FTC instead of acquired immunity against HBV, implying that anti-HBs levels were inadequate. After switching to CAB/RPV, the drugs no longer suppressed HBV. The exposure to reactivated HBV did not adequately boost the anti-HBs levels, which lead to breakthrough reactivation. Another potential explanation is a de novo reactivation of HBV independent of switching to CAB/RPV. However, we were unable to find documented cases of de novo HBV reactivation among persons on stable ART or with a stable immune status. Furthermore, whereas de novo HBV reactivation typically leads to severe hepatitis (*8*), our patient remained asymptomatic, with only a marginal elevation in transaminase levels.

E164V in the S region is known as a vaccine-escape mutation and is frequently identified in patients with occult HBV infection or de novo HBV reactivation (*9*,*10*). Cases of breakthrough infection are extremely rare worldwide, and it is not clear whether a single mutation is responsible for immune escape. In patients with occult HBV infection, escape mutations other than E164V are typically detected alongside other mutations (*10*,*11*).

HBV reactivation has been reported in patients with low CD4 counts (*12*,*13*). Cases have also been reported of HBV DNA detection at very low levels with negative HBsAg after switching to ART regimens without anti-HBV drugs (*14*). Our patient had a sufficient CD4 count for immunity and a history of anti-HBc and anti-HBs positivity, and still we found his HBV reactivated with re-emergence of HBsAg. We expect our patient will undergo the seroconversion of HBsAg again with ART containing F/TAF. Nonetheless, ART without tenofovir or lamivudine is not an option for this patient in the future.

## Conclusions

Below the cutoff value, the preventive effect of anti-HBs may not be sufficient to prevent reactivation. Vaccination may be beneficial for patients with isolated anti-HBc positivity, even if their anti-HBs levels have declined since their initial anti-HBs positivity was confirmed (*15*). For many years, ART containing tenofovir or lamivudine has been widely used worldwide; however, providers are increasingly using ART regimens that do not include anti-HBV drug. Healthcare providers must consider the potential scenario where the detection of anti-HBs might be a consequence of HBV suppression through ART containing anti-HBV drugs, and not indicative of a functional cure. Switching to ART regimens without anti-HBV drugs should be approached with caution.

Appendix Additional information about hepatitis B virus reactivation after switch to cabotegravir/rilpivirine in patient with low hepatitis B surface antibody.
