# Supplementary material for: Hepatitis B Virus Reactivation after Switch to Cabotegravir/Rilpivirine in Patient with Low Hepatitis B Surface Antibody
Source: Emerg Infect Dis. 2024 Aug;30(8):1668–71. doi: 10.3201/eid3008.240019 (PMC11286062; doi:10.3201/eid3008.240019)
Supplement: Appendix — Additional information about hepatitis B virus reactivation after switch to cabotegravir/rilpivirine in patient with low hepatitis B surface antibody. [file 24-0019-Techapp-s1.pdf]

Article DOI: https://doi.org/10.3201/eid3008.240019

EID cannot ensure accessibility for supplementary materials supplied by authors. Readers who have difficulty accessing supplementary content should contact the authors for assistance.

Hepatitis B Virus Reactivation After Switch to Cabotegravir/Rilpivirine in Patient with Low Hepatitis B Surface Antibody

Appendix

HBV-DNA extraction and PCR for whole genome sequencing

The HBV-DNA was extracted from the plasma using SMITEST EX-R&D (GS-J0201, Medical & Biologic Laboratories Co. Ltd, Nagoya, Japan). For polymerase chain reaction (PCR), KOD One PCR Master Mix (Toyobo) was used. The first PCR cycle program was 95°C 1 min, (98°C 10 s, 60°C 5 s, 68°C 20 s) × 30 cycles, 68°C 1 min, and 15°C forever. The second PCR cycle program was 95°C 1 min, (98°C 10 s, 60°C 5 s, 68°C 20 s) × 25 cycles, 68°C 1 min, and 15°C forever. The PCR product was purified using PCR Purification Kit (Qiagen, Hilden, Germany). The primer sets are detailed in the table below.

Appendix Table 1. Primer sets

Target	Primer name	Sequence	Fw/Rv	1st/2nd PCR
Full length 1	WA-L	ACTGTTCAAGGGTCCAAGCTGTGC	Fw	1st
	WA-R	AGCAAAAAGTTGCATGGTGCTGGT	Rv	1st
	WA-2L	GGTGGCTTTRGGRCATGGACAT	Fw	2nd
	WA-2R	CAGACCAATTTATGCCTACAGC	Rv	2nd
Full length 2	HBV_F7	ACGTCCTTTGTYTACGTCCCG	Fw	1st
	HBV_R7	GCGAGGCGAGGGAGTTCT	Rv	1st
	HBV_F4	TGCACTTCSCTTCACCTCTGCAC	Fw	2nd
	HBV_R5	GGAGGAGTGCGAATCCACACTCC	Rv	2nd

Whole genome sequence, alignment, and phylogenetic analysis

Sequencing of the full length of the HBV-DNA was performed using the direct (Sanger) sequencing method. Sequences obtained by direct sequencing were confirmed using ATGC-MAC Ver. 9.0.1 (GENETYX Corp., Tokyo, Japan) and aligned using GENETYX-MAC Ver. 22.0.1 (GENETYX Corp.). The text files of the alignments were converted to FASTA format using MAFFT Ver. 7. The primer sequences are as follows.

Appendix Table 2. Primers for HBV-DNA sequencing

Target	Primer name	Sequence
Full length 1	HBV B2466	GTAAAGTTTCCCACCTTATG
	HBV B2830	ATGCYGTAGCTCTTGTTCCC
	P4S	CAAGGTATGTTGCCCGTTTG
	HBV WA-2L	GGTGGCTTTRGGRCATGGACAT
	HBV WA-2R	CAGACCAATTTATGCCTACAGC
	HBV FA3L	CTGCTGGTGGCTCCAGTT
	HBV FA4L	GTATTGGGGGCCAAGTCTGT
	HBV FA2R	GGTATTGTGAGGADDYTTGTCAAC
	HBV 1260	GCCGATCCATACTGCGGAAC
Full length 2	HBV_F6	TGTCAACGACCGACCTTGAG
	HBV_R6	GAGAGTAACTCCACAGWAGCTCC
	HBV_F8	ACTGGGAGGAGYYGGGGGAG
	HBV_R8	CTCCACAGWAGCTCCAAATTCT

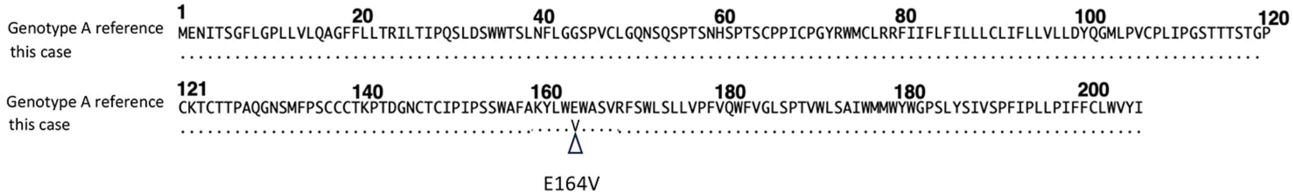

Appendix Figure. Alignment of the 226 amino acids of the HBV envelope is show. Mutational analysis revealed a missense mutation in the 164th amino acid of the S-region from the hydrophilic amino acid glutamic acid to the hydrophobic amino acid valine.
